# Predictive factors for the diagnosis of permanent congenital hypothyroidism and its temporal changes in Sergipe, Brazil – A real-life retrospective study

**DOI:** 10.20945/2359-3997000000579

**Published:** 2023-01-17

**Authors:** Hérika M. Gumes-Felix, Roberto J. R. Ramalho, Enaldo V. Melo, Diana M. Matos, Nelmo V. Menezes, Carla R. P. Oliveira, Viviane C. Campos, Elenilde G. Santos, Daniela da S. Marques, Brenda Vaz dos Santos, Bruna M. R. de Andrade, Manuel H. Aguiar-Oliveira

**Affiliations:** 1 Universidade Federal de Sergipe Divisão de Endocrinologia Programa de Pós-graduação em Ciências da Saúde Aracaju SE Brasil Divisão de Endocrinologia, Programa de Pós-graduação em Ciências da Saúde, Universidade Federal de Sergipe, Aracaju, SE, Brasil; 2 Universidade Federal de Sergipe Departamento de Medicina Aracaju SE Brasil Departamento de Medicina, Universidade Federal de Sergipe, Aracaju, SE, Brasil; 3 Universidade Federal de Sergipe Programa de Pós-graduação em Ciências da Saúde Departamento de Fonoaudiologia Aracaju SE Brasil Departamento de Fonoaudiologia, Programa de Pós-graduação em Ciências da Saúde, Universidade Federal de Sergipe, Aracaju, SE, Brasil

**Keywords:** Neonatal screening, congenital hypothyroidism, thyrotropin, thyroxine

## Abstract

**Objectives:**

Congenital hypothyroidism (CH) can be permanent (PCH) or transient (TCH). While the importance of thyroxine in myelination of the brain is undisputed, the benefits to neurodevelopmental outcomes of TCH treatment are controversial. Our objectives were to determine predictive factors for PCH and verify its prevalence changes over time.

**Subjects and methods:**

A total of 165 children were evaluated at 3 years of age to verify the diagnosis of PCH. 130 were submitted to a two-step cluster analysis, with the aim of grouping them into homogeneous clusters. The mean incidence of PCH and TCH was calculated from 2004 to 2010 and 2011 to 2015.

**Results:**

Sixty-six children were diagnosed with PCH, and 99 were diagnosed with TCH. Eighty-one percent of PCH children and all TCH children with thyroid imaging had glands in situ. Eighty children (61.5%) were in Cluster 1, 8 children (6.2%) were in Cluster 2 and 42 children (32.3%) were in Cluster 3. No children had PCH in Cluster 1, while 87.5% of children in Cluster 2 and all children in Cluster 3 had PCH. The most important predictor for PCH was the initial serum TSH, which was marginally higher in importance than the blood spot TSH, followed by the initial serum free T4. The mean incidence of PCH (odds ratio: 1.95, 95% CI 1.36 to 2.95, p < 0.0001) and TCH (odds ratio 1.33, 95%, CI 1.02 to 1.77, p = 0,038) increased over time.

**Conclusions:**

The most important PCH predictors are the initial serum TSH and the blood spot TSH. The mean incidence of both PCH and TCH in our series increased.

## INTRODUCTION

Congenital hypothyroidism (CH) can be permanent (PCH) due to thyroid dysgenesis or dyshormonogenesis or, in up to 35% of cases, transient (TCH) ( 1 ). Although gene mutations of DUOX2 and TSH-R have been described in TCH ( 2 – 4 ), most of these cases do not have a defined cause, with the iodine intake, the decrease in the cutoff levels of the neo-natal screening programs (NSP), and the time of blood spot collections being the factors initially suggested for the occurrence of nongenetic TCH cases ( 5 ).

A neonatal screening program (NSP) for CH (CH-NSP) is one of the most notable milestones in public health. Since its inauguration by Dussault ( 6 ) in Quebec-Canada in 1972, severe cases of CH with irreversible mental retardation have been abolished in countries with wide coverage CH-NSP. Currently, the focus is on ensuring the maximum neuromotor and intellectual development of those affected. To achieve this, an anticipation of the age of treatment and a reduction of cutoff points have been used worldwide. However, half a century later, some issues in the CH-NSP remain unresolved. Among these, the overtreatment of transient CH cases may have medical consequences and generate unnecessary concerns in affected families ( 7 ). The importance of thyroxine in myelination of the infant brain is indisputable, as has been recently shown in MRI studies ( 8 ). Although the reason to detect severe CH is unequivocal, the benefit for neurodevelopmental outcomes when treating babies with modest TSH elevations and borderline free T4 (FT4) concentrations still generates discussions, with some studies showing a relationship between academic performance and neonatal TSH ( 9 ), while others do not ( 10 ).

Another intriguing issue is the worldwide increased incidence of CH ( 11 ), especially with glands *in situ* ( 12 ). The reduction of the neonatal TSH cutoff values and the increased survival of premature and low birth weight children can contribute to this increase ( 13 ). However, the reasons for this increase remain unclear, although genetic, epigenetic, and environmental factors have been proposed ( 14 – 18 ).

The Brazilian Health Ministry Policy established in 2001 the University Hospital of the Federal University of Sergipe (HU-UFS) as the unique entity to perform the NSP in the State of Sergipe, which has used the same cutoff point of neonatal bloodspot TSH (5.2 μU/mL) since its implementation. This allows an appropriate comparison of different time periods ( 19 – 21 ). In a previous paper, we found that among children with serum initial TSH levels greater than 10 μU/mL (more than two-thirds of the cases had PCH), 13.5% had TCH. In children with initial serum TSH levels greater than 4.2 μU/mL and less than or equal to 10 μU/m, 13.8% had PCH, while 58.1% had TCH ( 21 ). The main objective of the present study was to assess predictive factors for the diagnosis of permanent congenital hypothyroidism in a newborn with abnormal neonatal blood spot TSH. An additional objective is to verify whether the incidence of permanent and transient CH has changed in the periods from 2004 to 2010 and from 2011 to 2015.

## SUBJECTS AND METHODS

A descriptive, analytical, and retrospective study was carried out in children with abnormal screening tests in the CH-NSP in the state of Sergipe from 2011 to 2015 and followed at the outpatient clinics of the University Hospital of Federal University of Sergipe. According to our protocol, all children with abnormal neonatal TSH (TSH ≥ 5.2 µU/mL) were summoned for collection of TSH and total T4 and free t4 in blood: venous. If serum TSH was less than or equal to 4.2 µU/mL, with normal free T4, in two consecutive measurements, the patient was considered as normal and excluded. If serum TSH was between 4.21 and 10 µU/mL with normal free T4, the child was classified as having hyperthyrotropinemia. In those with serum TSH ≥ 10 µU/mL or between 4.21 and 10 µU/mL with low free T4, treatment was started, and children were referred to the pediatric endocrinology outpatient clinic. At the age of three years, those who had serum TSH greater than 10.0 µU/mL and who required high doses of levothyroxine were classified as PCH. In children requiring low doses and with no established etiology for CH, levothyroxine therapy was interrupted for a period of 30 days to measure TSH and free T4 to assess the permanence or transience of the condition.

The inclusion criteria were neonatal TSH (measured by the immunofluorimetric method, AutoDELFIA, Perkin-Elmer, Life-Sciences, Turku, Finland) higher than 5.2 µU/mL and confirmed serum TSH above 4.2 µU/mL ( 21 ). The exclusion criteria were absence of medical records in the outpatient facility of the University Hospital; loss of follow-up before the final diagnosis; and children with a final diagnosis of hyperthyrotropinaemia after the measurement of fT4. This is a permanent condition, with uncertain physiological impact, defined in children without levothyroxine replacement therapy, with serum TSH between 4.2 µU/mL and 10 µU/mL, but with normal fT4. The study variables were birth date, sex, origin, prematurity, neonatal blood spot TSH, initial serum TSH and free T4. PCH was defined by serum TSH greater than 10 µU/mL, regardless of fT4 values, or using levothyroxine, or without levothyroxine use with serum TSH between 4.2 µU/mL and 10 µU/mL, but free T4 less than 0.79 ng/dL. TCH was defined as a child initially screened as CH who normalized in the follow-up after levothyroxine withdrawal (serum TSH less than or equal to 4.2 µU/mL, free T4 greater than or equal to 0.79 ng/dL). The normal reference value for free T4 was ≥ 0.79 ng/dL.

Out of 172,547 newborns born from 2011 to 2015 (Sergipe Datasus-MS), 140,325 were screened by NSP-SE (81.32% coverage), of which 767 (0.54%) presented an abnormal neonatal bloodspot test (neonatal TSH greater than 5.2 µU/mL). Fifty-eight (7.5%) children were not located by the social service for the confirmatory serum test, and 391 (50.97%) children were excluded due to serum initial TSH lower than 4.2 µU/mL. The medical records of 315 remaining children were searched to assess follow-up and final diagnosis. Children whose medical records were missing or lost to follow-up before final diagnosis were excluded from the study. One hundred and sixty-five children (21.5% of altered neonatal bloodspot tests) were eligible for this study. In children in whom there was doubt about the permanent or temporary condition of CH, levothyroxine was suspended at age three to assess whether CH persisted after this suspension. Imaging techniques (ultrasound and/or thyroid scintigraphy) were performed in 88 children. These images were summarized in three groups: gland *in situ* , hyperplasia, and dysgenesis (agenesia and ectopic thyroid). The IRB Committee of the Federal University of Sergipe approved the research project under the number CAAE 43123021.0.0000.5546.

### Statistical analysis

Shapiro-Wilk and Kolmogorov-Smirnov tests were used to verify the normality of the variables. The continuous variables exhibited a nonnormal distribution, and they were expressed as the median (interquartile distance). Differences between the PCH and TCH groups were analyzed by the Mann-Whitney test. Categorical variables were expressed as absolute frequencies and percentages and were compared by Fisher's exact test. The comparison of variables among the three thyroid imaging groups was performed by the independent-samples Kruskal-Wallis test, with pairwise comparisons.

In this paper, we used cluster analysis, which is a multivariate analysis that allows the identification of groups with homogeneous characteristics and can be used when there are at least three numerical variables. This technique disaggregates a set of objects into smaller subsets (clusters) according to their characteristics (variables). By using mathematical distance calculations, it is possible to assign a measure of proximity (similarity) between each object and clusters. Accordingly, the clusters formed so that the distances between the members of a cluster are minimal and the distances between the clusters are maximal.

The two-step cluster analysis method ( 22 ) was used with categorical (PCH diagnosis) and continuous (neonatal blood spot TSH, serum initial TSH and serum initial fT4) variables. The log-likelihood method was used for distance measurements. The number of clusters to be formed was not specified in advance. We estimated the silhouette of cohesion and separation measures, a measure for the overall suitability of the found cluster structure. It ranges from -1 to 1 and is thus defined as follows: less than 0.25: no substantial structure; 0.26 to 0.50: weak structure; 0.51 to 0.70: fair structure; and 0.71 to 1.0: strong structure. Differences in characteristics between clusters were compared by the independent-samples Kruskal-Wallis test, with pairwise comparisons.

The mean incidence of each final condition was calculated by WinPepi version 4.0, dividing the number of affected children with PCH or TCH by the number of children screened during the period. The 95% confidence interval (CI): 95% CI and odds ratio (OR) were also registered. Statistical analysis was performed using IBM SPSS (Statistical Package for the Social Sciences), version 23.0, Chicago, IL, USA). The accepted level of significance was less than 0.05, and all tests were two-tailed.

## RESULTS

Table 1 shows the possible predictive factors for the diagnosis of PCH in 66 children or TCH in 99 children. While the age of treatment onset and sex were similar, the levels of neonatal blood spot and serum initial TSH were higher and the levels of fT4 were lower in PCH than in TCH (p < 0.0001, in all comparisons).

**Table 1:** Possible predictive factors to the diagnosis of permanent (PCH) in 66 children, or transient (TCH) in 99 children, with congenital hypothyroidism

	PCH	TCH	p
Age at treatment onset (days)	54.0 (33)	55 (40)	0.371
Gender male n (%)	29 (43.9)	57 (57.5)	0.112
Bloodspot TSH (µU/mL)	9.10 (42.54)	6.42 (1.76)	<0.0001
Initial serum TSH (µU/mL)	13.0 (192.62)	5.54 (1.89)	<0.0001
Initial serum FT4 (ng/dL)	0.99 (0.3)	1.14 (0.15)	<0.0001

Values expressed as median (interquartile range), unless for gender expressed in n (%). Mann-Whitney test.

Table 2 shows the final diagnosis of PCH or TCH according to the increasing concentrations of serum initial TSH. Two-thirds of PCH and most HCT cases had an initial serum TSH between 4.2 and 20 µU/mL. Conversely, one-third of PCH and only 8% of TCH cases had initial serum TSH greater than 41 µU/mL This distribution of frequencies was highly different (p < 0.0001, Fisher's exact test).

**Table 2:** Final diagnosis of permanent (PCH) or transient (TCH) in according to the increasing concentrations of serum diagnosis TSH (µU/mL)

Diagnosis		4,2 to 20	21 to 40	41 to 100	>100	Total
PCH	n (%)	40 (61)	3 (4.5)	5 (7.5)	18 (27)	66 (100)
TCH	n (%)	92 (93)	0 (0.0)	1 (1.0)	6 (6.0)	99 (100)

Values expressed as n (%). P < 0.0001, Fisher's exact test.

Table 3 shows the final diagnosis of PCH or transient TCH according to the decreasing concentrations of serum initial fT4. Half of the PCH and most THC cases had an initial ft4 between 0.79 and 1.16 ng/dL. Conversely, a third of PCH and a minority of TCH cases had an initial ft4 lower than 0.4 ng/dL. This distribution of frequencies was highly different (p < 0.0001, Fisher's exact test).

**Table 3:** Final diagnosis of permanent (PCH) or transient (TCH) in according to the decreasing concentrations of serum free T4 (ng/dL)

Final diagnosis		0.79 to 1.16	0.4 to 0.78	<0.4	Total
PHC	n (%)	30 (45.4)	18 (27.3)	18 (27.3)	66 (100)
THC	n (%)	87 (87.9)	10 (10.1)	2 (2.0)	99 (100)

Values expressed as n (%), P < 0.0001, Fisher's exact test.

Out of 58 PCH cases, 54 (93.1%) were full-term, and four (6.9%) were preterm newborns. Out of 88 TCH cases, 81 (92%) were full-term, and seven (8.0%) were preterm newborns with similar frequency distributions (p = 0.541, Fisher's exact test).

Table 4 shows the thyroid morphology in 58 children with PCH and 30 with TCH. Most cases of PCH (81%) and all cases of TCH had glands *in situ* . This distribution of frequencies was different (p = 0.034, Fisher's exact test).

**Table 4:** Thyroid morphology in 58 children with permanent (PCH) and 30 with transient (TCH) congenital hypothyroidism

Final diagnosis		*In situ*	Hyperplasia	Dysgenesis	Total
PCH	n (%)	47 (81.0)	5 (8.6)	6 (10.4)	58 (65.9)
TCH	n (%)	30 (100.0)	0 (0)	0 (0)	30 (34.1)

Values expressed as n (%), p: 0.034 Fisher's exact test.

Table 5 shows the hormonal data according to thyroid morphology in 58 PCH and 30 TCH children. While TSH in blood spots in the serum increased from the gland *in situ* to hyperplasia and dysgenesis, the levels of fT4 were higher in the gland *in situ* in comparison to the other categories.

**Table 5:** Hormonal date in according to thyroid morphology in 58 children with permanent (PCH) and 30 with transient (TCH) congenital hypothyroidism

	Gland *in situ*		Hyperplasia	Dysgenesis	P
N	77	5		6	
Bloodspot TSH (µU/mL)	7.5 (5.2)	101.4 (87.0)		162.7 (265.5)	<0.0001
Initial serum TSH (µU/mL)	8.7 (16.4)	223.0 (415.1)		333.0 (594.6)	0.001
Initial serum fT4 (ng/dL)	1.0 (0.3)	0.4 (0.7)		0.8 (0.12)	0.039

Values expressed as median (interquartile range) and compared by the Kruskal-Wallis's test. The pairwise comparisons showed for neonatal blood spot TSH that gland *in situ* x hyperplasia: p = 0.045, situ x dysgenesis: p = 0.001. For serum initial TSH, gland *in situ* x dysgenesis: p = 0.005.

Table 6 shows the characteristics of the three discriminated clusters in 130 screened children and the number of permanent congenital hypothyroidism (PCH) cases in each cluster. Eighty (61.5%) of the newborns were located in Cluster 1, 8 (6.2%) in Cluster 2, and 42 (32.3%) in Cluster 3. No children had PCH in Cluster 1, while 87.5% of children in Cluster 2 and all children in Cluster 3 had PCH. The structure of this analysis was strong, with a medium silhouette value of 0.9. The analysis of the importance of predictors for PCH revealed that serum initial TSH was the most important (predictor importance, 1.00) and marginally more important than the neonatal blood spot TSH (predictor importance 0.97), followed by the serum initial fT4 (predictor importance 0.07).

**Table 6:** Characteristics of the clusters in 130 screened children and the number of permanent congenital hypothyroidism (PCH) in each cluster

Characteristics	Cluster 1 (n = 80)	Cluster 2 (n = 8)	Cluster 3 (n = 42)	P
Age of treatment (days)	53 (39)	36 (19)	55 (30)	0.113
Gender Male n (%)	50 (62.5)	4 (50.0)	21 (50.0)	0.376
Blood spot TSH	6.3 (1.6)	96.1 (109.2)	7.4 (4.7)	<0.0001
Initial serum TSH	5.5 (1.7)	238.0 (259.0)	8.7 (10.1)	<0.0001
Initial serum fT4	1.1 (0.1)	0.4 (0.3)	1.0 (0.2)	<0.0001
PCH in Cluster n (%)	0 (0)	7 (87.5)	42 (100)	<0.0001

Values expressed as median (interquartile range) and compared by the Kruskal-Wallis's test, unless for gender and PCH cases in Cluster expressed in n (%). The pairwise comparisons showed for neonatal blood spot TSH that Cluster 1 x Cluster 2: p < 0.0001, Cluster 2 x Cluster 3: p = 0.003, Cluster 1 x Cluster 3: p = 0.031. For serum initial TSH, Cluster 2 x Cluster 1: p < 0.0001, Cluster 2 x Cluster 3: p = 0.019, Cluster 1 x Cluster 3: p < 0.0001. For serum initial fT4, Cluster 1 x Cluster 2: p < 0.0001, Cluster 1 x Cluster 3: p < 0.0001.

From 2004 to 2010, out of 193,794 screened newborns, 46 had a diagnosis of PCH, with a mean incidence of 1:4,166. From 2011 to 2015, out of 140,325 screened newborns, 66 had a diagnosis of PCH, with a mean incidence of 1:2,126 (odds ratio: 1.95, 95% CI 1.36 to 2.95, p < 0.0001). From 2004 to 2010, out of 193,794 screened newborns, 102 had a diagnosis of TCH, with a mean incidence of 1:1,900. From 2011 to 2015, out of 140,325 screened newborns, 99 had a diagnosis of TCH, with a mean incidence of 1:1,1417 (odds ratio: 1.33, 95% CI 1.02 to 1.77, p = 0.038).

## DISCUSSION

CH is a different disease after 50 years of CH-NSP. Before neonatal screening, it was a multisystem disease, with dermatologic, hematologic, cardiopulmonary, intestinal, and brain manifestations, marked hypometabolism, severe short stature, sometimes inability to maintain a standing position, pubertal delay, and marked mental retardation. Late treatment with l-thyroxine improved metabolic and systemic status, but the mental deficit was irreversible, sometimes with worsening of preexisting seizures. Currently, CH is a disease with minimal brain involvement and some intellectual and fine motor deficits, and the focus is on ensuring maximum neuromotor and intellectual development of these patients. However, even today, the functioning of a family with a patient with sequelae of CH may be compromised. Diagnosing and correctly treating CH children in a timely ( 19 ) manner represents a significant benefit for the patient, his or her family, and the community, reducing social, emotional, and financial costs, including higher-cost special schools ( 8 ). Treatment should be started with a daily dose of levothyroxine of 10 to 15 mcg/kg, ideally starting prior to the 14th day after birth, but the overtreatment of TCH cases may have medical consequences and generate unnecessary concerns in affected families ( 7 ), and the neurodevelopmental benefits in these children remain controversial ( 9 , 10 ).

Here, we found that while the age of treatment onset and sex were similar in PCH and TCH, the levels of neonatal blood spot and serum initial TSH were higher and the levels of fT4 were lower in PCH than in TCH. When we analyzed the diagnosis of PCH or transient (TCH) according to the increasing concentrations of serum initial TSH, a highly different distribution of frequencies was found. Two-thirds of PCH and most HCT cases had serum initial TSH between 4.2 and 20 µU/mL. Conversely, one-third of PCH and only 8% of TCH cases had an initial serum TSH greater than 41 µU/mL. When we analyzed the prevalence of PCH or TCH according to the decreasing concentrations of serum initial ft4, a highly different distribution of frequencies was also found. Half of the PCH and most THC cases had an initial ft4 between 0.79 and 1.16 ng/dL. Conversely, a third of PCH and a minority of TCH cases had an initial ft4 less than 0.4 ng/dL. These data agree with an Irish study, which suggested that higher neonatal blood spot and initial serum TSH concentrations and lower serum initial fT4 levels were associated with an increased likelihood of permanent CH. However, in that study, it was not possible to predict the outcome with certainty because significant variation was found. In this study, PCH was present in one-third of infants with serum TSH concentrations between 8 and 20 mU/L, and TCH was observed in one-fifth of infants with serum TSH concentrations above 100 mU/L ( 17 )

From a practical point of view, in clinical practice, it is very important, when the physician receives a newborn with altered neonatal TSH results, to know which factors can be predictors of the diagnosis of PCH versus TCH. To this end, a two-step cluster analysis method was used, with categorical (PCH diagnosis) and continuous (neonatal blood spot TSH, serum initial TSH and serum initial fT4) variables. Three clusters were identified, and the number of PCHs in each cluster was measured. Eighty screened children (61.5%) were in Cluster 1, 8 (6.2%) were in Cluster 2, and 42 (32.3%) were in Cluster 3. No children had PCH in Cluster 1, while 87.5% of children in Cluster 2 and all children in Cluster 3 had PCH. The analysis of the importance of prediction for PCH revealed that the initial serum TSH was the most important and was marginally more important than the neonatal blood spot TSH, followed by initial serum free T4. Therefore, the serum initial TSH is the best predictor of the diagnosis of PCH.

Most cases of PCH and all cases of TCH had glands *in situ* . This finding agrees with several previously published reports ( 12 , 17 , 23 ). The etiology of CH with glands *in situ* remains unclear, and the factors that determine its clinical diversity are unknown. The possible effects of toxic environmental agents on the fetal and neonatal thyroid gland are currently being studied in several scenarios ( 23 – 25 ).

Another important finding of the current work is the significant increase of the incidence of PCH and TCH comparing the 2004-2010 and the 2011-2015 periods in the same region, without demographic changes, with the same iodine intake, the same screening procedure, and the same cutoff point in both periods ( 21 ). A similar increase was also demonstrated in Ireland over a period of 37 years. The authors suggest that other potential causes, such as iodine deficiency or excess, and environmental factors need to be considered ( 17 ). Several pollutants have been studied as potentially toxic to fetal and neonatal thyroid that might impact the transcription of genes involved in thyroid gland development, such as perchlorate ( 18 ), halogenated organochlorines, pesticides, polychlorinated biphenyls and polybrominated diethyl ethers ( 25 – 27 ), fungicides ( 28 ), bisphenol ( 29 ) and nitrogen oxide ( 30 ). A Scottish study using space-time clustering suggested the involvement of a spatially varying and transient environmental agent or agents. Such agents could include pollutants or pesticides, which would be expected to occur in more rural communities ( 24 ). Brazil is a great consumer of pesticides ( 31 ), but we are not aware of any influence of these environmental factors on the incidence of CH in Sergipe. The increase in CH incidence seems widespread, as was also demonstrated in the past decade in Xiamen, China, where the incidence of CH has increased, due to an increase in the incidence of both PCH and TCH, mainly in those with glands *in situ* ( 32 ). The authors suggested potential causes of CH, such as iodine deficiency or excess and environmental factors, and genes susceptible to CH ( 32 ).

Some factors can explain the increase in the incidence of CH, such as the reduction in the cutoff point and the demographic composition of the population. In Italy, the reduction of TSH cutoff to 6 mU/L from cutoffs above or equal to 6 and lower than 7 mU/L, above or equal to 7 and lower than 10, and equal to or above 10 mU/L allowed the identification of one-fifth of newborns with confirmed out-of-range TSH, otherwise not recognized by the previously employed TSH cutoff ( 33 ). Our CH-NSP uses the same cutoff level since February 2003 ( 19 ); therefore, this is not the factor for the increase in the incidence of CH. Another possible factor for changes in CH could be the demographic composition. Hispanics and Asians are reported to have the highest CH incidences (1:1,600 and 1:2,380, respectively) ( 34 ). Sixty percent of the population of northeastern Brazil is of European origin ( 35 ), and Sergipe is one of the regions where the Portuguese began the colonization of Brazil. However, the composition of the population did not change in the twelve years of this study, and therefore, demographics are not a reason for the increase in CH incidence in Sergipe.

Evidence from different screening programs indicated that the rate of CH was higher in preterm and low birth weight infants than in those born at term. Incomplete development of the hypothalamic-pituitary axis in preterm infants and low birth weight infants may result in a delayed rise in TSH. To bypass this problem, the recommended strategies are using both TSH and fT4 for screening preterm infants and a second screening at two, four, six and ten weeks of life ( 36 – 38 ). Although we did not use any of these strategies in our CH-NSP, the percentage of preterm newborns was similar in PCH and TCH, which does not seem to influence the increase in the incidence of both PCH and TCH in the two periods of the study. A recent Canadian study concluded that, at the time of diagnosis, in the absence of known thyroid dysgenesis, there are no clear predictors of TCH, although thyroxine dose, an increase in serum TSH above the reference range during treatment, neonatal blood TSH and a history of maternal thyroid disease appear to predict TCH in some way ( 39 ). This work differs from ours, as only one-third of the cases of PCH presented glands *in situ* , while glands *in situ* were present in our study in 81% of the cases of PCH.

The limitations of our study include the possible incomplete quality of medical records, the loss of newborns after neonatal TSH collection and the loss of follow-up before the final diagnosis, which reduced the number of children studied.

In conclusion, the two-step cluster analysis discriminated three clusters in children with an altered neonatal TSH blood spot. No children had PCH in Cluster 1, while 87.5 percent of children in Cluster 2 and all children in Cluster 3 had PCH. The most important predictor of PCH was the initial serum TSH, which was marginally higher than the blood spot TSH. The mean incidence of both PCH and TCH increased from 2004 to 2010 and from 2011 to 2015. The reason for this increase remains unclear.
